# Hematemesis as Initial Presentation in a 10-Week-Old Infant with Eosinophilic Gastroenteritis

**DOI:** 10.1155/2017/2391417

**Published:** 2017-02-16

**Authors:** Varun Shetty, Kayla E. Daniel, Anil Kesavan

**Affiliations:** ^1^Department of Medicine-Pediatrics, Rush University Medical Center, 1645 W. Jackson Blvd., Suite 215, Chicago, IL 60612, USA; ^2^Department of Pediatrics, Nationwide Children's Hospital, 700 Children's Dr, Columbus, OH 43205, USA; ^3^Section of Pediatric Gastroenterology, Rush University Medical Center, Professional Building, 1725 W. Harrison Street, Suite 710, Chicago, IL 60612, USA

## Abstract

Eosinophilic gastroenteritis is a rare condition characterized by eosinophilic inflammation in the gastrointestinal tract resulting in a variety of gastrointestinal symptoms. There is currently a dearth of information on this topic in the pediatric literature, as very few cases have been reported. In this report, we present a case of eosinophilic gastroenteritis in a 10-week-old patient with initial presenting symptom of hematemesis. To our knowledge, this is the youngest case reported in the literature and is unique in its initial presentation.

## 1. Introduction

First described in 1937 by Kaijser et al. as an allergic disease of the gut, eosinophilic gastrointestinal disorders are a spectrum of clinical diseases that present with varying degrees of eosinophilic infiltration of the gastrointestinal tract in the absence of other known causes of tissue eosinophilia [1]. This series of diseases includes eosinophilic esophagitis, eosinophilic gastritis, eosinophilic gastroenteritis, eosinophilic enteritis, and eosinophilic colitis. While eosinophilic esophagitis is well characterized in the pediatric population, the other eosinophilic gastrointestinal disorders are rare and not fully understood. Symptoms include abdominal pain, nausea, vomiting, poor appetite, weight loss, and diarrhea [2]. These symptoms typically present between the third and fifth decades of life [3]. Findings on endoscopic exam are variable, and several treatment modalities have been proposed.

## 2. Case Presentation

A 10-week-old full-term female infant with no significant past medical history presented with a one-day history of projectile hematemesis. She was well appearing on exam with stable vital signs and no evidence of hemodynamic instability. Laboratory studies were significant for a normocytic, hypochromic anemia (hemoglobin 7.4 g/dL on admission) with the presence of target cells on red blood cell morphology suggesting a hemoglobinopathy such as beta thalassemia or alpha thalassemia. Abdominal X-ray and PT/INR were normal. She was started on an H2 receptor antagonist and remained asymptomatic with no further episodes of hematemesis while hospitalized. It was felt that her symptoms were due to a possible hemoglobinopathy in conjunction with gastritis or a Mallory-Weiss tear. She was discharged home to continue an H2 receptor antagonist with close follow-up.

**Figure 1:**
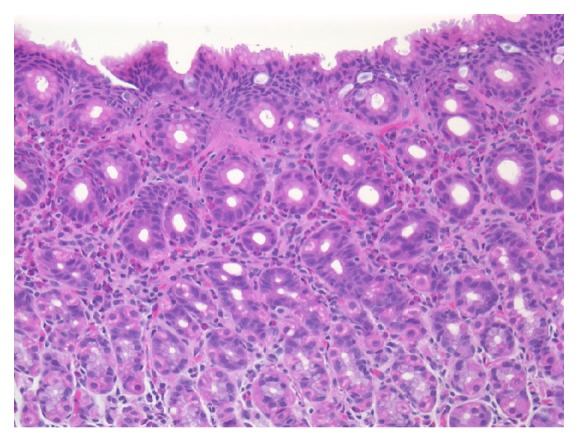
Moderate to severe chronic gastritis with focally increased intraepithelial eosinophils.

She was readmitted approximately one week later with similar complaints. She had been doing well until the day of admission with normal feeding and no fever, diarrhea, constipation, melena, hematochezia, or upper respiratory infection symptoms. Laboratory results were significant for hemoglobin of 6.9 g/dL and thrombocytosis. A complete metabolic profile, lipase, and PT/INR were normal. She was started on a proton pump inhibitor and an esophagogastroduodenoscopy (EGD) and flexible sigmoidoscopy were performed. EGD revealed a single gastric antral erosion; the remainder of the stomach, duodenum, and esophagus were normal in appearance. No abnormalities were seen on flexible sigmoidoscopy. Multiple areas of gastric antral and antral-corpus junction mucosa demonstrated moderate to severe chronic gastritis with focally increased eosinophils consistent with eosinophilic gastritis (Figure 1). The esophagus, duodenum, sigmoid colon, and rectum were histologically normal with no presence of disease. The patient was subsequently started on an elemental formula. She remained clinically well with a stable hemoglobin throughout admission. Further hematologic evaluation was undertaken to evaluate the presence of a hemoglobinopathy. These studies were normal; no evidence of any hematologic disease was found. She was advised to continue the proton pump inhibitor and elemental formula, with close follow-up after discharge. She did not continue the proton pump inhibitor after discharge but remained on an elemental formula. She has not had any recurrence of hematemesis and has remained well with excellent weight gain and growth.

## 3. Discussion

Eosinophilic gastroenteritis (EG) is a disease characterized by eosinophilic inflammation involving predominately the stomach and proximal small intestine [1]. The entire intestinal tract from esophagus to colon, however, can be affected in patients with EG. The estimated incidence of EG is cited as between 1 and 20 cases per 100,000 patients [1]. Most of the current literature describes EG in adults; reports of the clinical or histopathological features of EG in children are rare [4]. The pathogenesis of EG is not well understood though a delayed-type hypersensitivity reaction is suspected. Recent studies have noted the role of T-helper cytokines and other mediators such as interleukin-5 and eotaxin-1 in peripheral blood mononuclear cells of EG patients, consistent with in vivo activation by food allergens [5, 6].

The epidemiology, disease course, and histopathological features of EG are highly variable. Common epidemiologic characteristics include higher incidence among adult males and those with a personal history of atopy. Tissue eosinophilia, peripheral eosinophilia, and heightened IgE levels are commonly reported. Tissue eosinophilia infiltration can be seen in mucosa, muscularis, or serosa [7]. Mucosal involvement may produce nausea, vomiting, diarrhea, abdominal pain, protein losing enteropathy, or malabsorption [2]. GI bleeding has been reported as a rare manifestation of this disease [8]. Involvement of the muscularis may produce obstruction (especially of the pylorus), whereas serosal activity produces eosinophilic ascites. In contrast with eosinophilic esophagitis (EoE), stricture formation is not a common feature of EG.

There are no consensus guidelines for diagnosis of EG. Diagnosis is generally made if eosinophilic infiltration of the gastrointestinal tract is seen on biopsy and/or eosinophilic ascitic fluid in patients with typical symptom in the absence of other causes of gut eosinophilia. The most common gross endoscopic finding is the presence of gastric pseudopolyps, although endoscopic findings are highly variable [4]. Other findings include erythema, erosions, and lymphonodular hyperplasia occurring in the antrum, fundus, and pyloric regions of the stomach.

The disease course of EG varies in the literature. A subset of cases respond to dietary elimination or elemental diet, although further studies are needed to prove efficacy [5, 9]. Pharmacologic therapy with histamine blockers, mast cell stabilizers, or leukotriene inhibitors is also commonly used [10]. Patients will often require systemic or enteral administration of corticosteroids and most progress to chronic disease course marked by severe malabsorption and malnutrition, highlighting the need for new approaches to treatment [11]. Recent efforts have included monoclonal antibody therapies against IgE and IL-5 [12].

## 4. Conclusion

Eosinophilic gastroenteritis is a rare disease in the pediatric and general population with a high variety of presenting symptoms and endoscopic features. To our knowledge, our case highlights the youngest reported diagnosis of EG, presenting with the uncommon presentation of hematemesis. While rare, EG should be considered in children of any age with a presenting symptom of hematemesis. Elemental formula can be an effective treatment in this condition.

## Abbreviations

EG: Eosinophilic gastroenteritis
EGD: Esophagogastroduodenoscopy
EoE: Eosinophilic esophagitis.
